# Hysterica Epileptica with Unusual Complications

**Published:** 1878-02-20

**Authors:** John Coston

**Affiliations:** Alabama


### HYSTERICA EPILEPTICA WITH UNUSUAL COMPLICATIONS.

By John Coston, M.D., of Alabama.

I submit to your disposal the following history ef a case under my observation for some time past, hoping it may prove beneficial to the afflicted, and, perhaps, call forth some thought and investigation by the profession ou the important subject of the Neuroses.

### HISTORY OF PATIENT.

Mrs. B., aged 19, was married at 16. She is small of stature, weighs about 100 pounds; her health was good, in the main, before marriage, but even prior to the appearance of the menses she had fluor albus, which has persisted up to the present. Her monthly courses had been on her regularly nine times before she married, and they continued regularly for four months after marriage, when she became pregnant. She went through her term, and brought forth in due time, and recovered as well as usual. Her courses came on at three months, and continued regular up to six months, when they were again interrupted by conception. She passed her second pregnancy and confinement very well: this last confinement occurred December 25th, 1876. The last child died at four weeks old.

### HISTORY OF THE CASE AS GIVEN BY THE PATIENT.

In less than two months after she was married she began to suffer, as she expresses it, "with flashes of burning pain," starting in the left ankle, about the external maleolus, and running up toward the knee, the pain continuing but a moment, and recurring every few days. The pain increased regularly in intensity and in frequency and in the extent of its range, so it soon began to run to the knee and then above it, and afterwards to the hip, and finally, in the latter part of her second pregnancy, it reached the head and on two occasions, a short time before her last delivery, it affected her mind and suspended respiration, which it has continued to do occasionally ever since. The spells last from two to five minutes, and they had increased in frequency until she was having from four to seven spells in the twenty-four hours by July 19, 1877, when I first saw her.

She then presented the following conditions: Her general appearance indicated pretty good health; she reports appetite good; bowels regular; the tongue clean; the lungs, liver, heart and spine seem to be healthy, save a slight tenderness of spine in sacral region; uterus quite tender, slightly enlarged and slightly ulcerated, depressed a little, and seems to be inclined to retrovert; diffused tenderness through the abdomen; severe vaginismus, so as to preclude copulation for months—indeed, it has always given her pain; numerous tender places along the crest of the ilium, about the sacrum; the sciatic notch, behind the trochanter magor, along the track of the sciatic nerve in the thigh, head of femur, etc., etc.

From the time she feels the pain, it is not usually more than half a minute till it reaches the head. It then draws it forward and to the left, for the left side draws worse than the right. The pains sometimes abort, or fail to reach the head, but when they do reach the head, and pass off, then she is for some minutes quite deranged, picking and pulling at the clothing, and giviug but little heed to anything that is said; then there follows a general rumbling of the bowels.

There have been several changes in the case during the time of my observation. Uterine troubles greatly relieved; deep-seated tenderness in abdomen better, but a greater degree of tenderness of the surface has ensued. Spinal tenderness has grown worse, the spells have decreased to three in the last fifteen days; the menses have not yet appeared.

I do not consider it necessary to detail the treatment. It has consisted of alteratives, tonics, antispasmodics, anodynes and counter-irritants, baths, etc., with local applications to uterus and vagina.

The points that most prominently present themselves to my mind are—

1. That fluor albus existed before the menses ever appeared.

2. That the spells came on very soon after marriage, and while the courses were regular.

3. That the spells were not in any way influenced by the changes incident to two pregnancies, deliveries and lactations.

4. The explosive character of the sciatic pain.

5. That the sciatic pain existed two years before it assumed its present epileptical form.

Now, will those better acquainted with this class of diseases do me the kindness to give me their opinions on the etiology, diagnosis and treatment of the case through the RECORD, that we may have light on this somewhat singular affection?

[The above case presents a remarkable illustration of the power of what is styled reflex irritation in the production of varied and singular phases of diseased action at remote points of the system. The name or caption to the article is our own—our correspondent having failed to give one. Perhaps we are incorrect. Let some one answer his inquiries.—EDITOR RECORD.]

